# The Sulfoquinovosyltransferase-like Enzyme SQD2.2 is Involved in Flavonoid Glycosylation, Regulating Sugar Metabolism and Seed Setting in Rice

**DOI:** 10.1038/s41598-017-04002-2

**Published:** 2017-07-05

**Authors:** Xinqiao Zhan, Qingwen Shen, Xuemin Wang, Yueyun Hong

**Affiliations:** National Key Laboratory of Crop Genetic Improvement, Huazhong Agricultural University, Wuhan, 430070 China

## Abstract

Seed setting is an important trait that contributes to seed yield and relies greatly on starch accumulation. In this study, a sulfoquinovosyl transferase-like protein, designated as SQD2.2 involved in seed setting and flavonoid accumulation, was identified and characterized in rice. Rice SQD2.2 is localized to the cytoplasm, and the *SQD2.2* transcript was highest in leaves. Rice *SQD2.2-*overexpressing (OE) plants exhibited a decreased seed setting rate and diminished tiller number simultaneously with an increased glycosidic flavonoid level compared with wild-type (WT) plants. SQD2.2 catalyzes the glycosylation of apigenin to produce apigenin 7-O-glucoside using uridine diphosphate-glucose (UDPG) as a sugar donor, but it failed to compensate for sulfoquinovosyldiacylglycerol (SQDG) synthesis in the Arabidopsis *sqd2* mutant. Furthermore, apigenin 7-O-glucoside inhibited starch synthase (SS) activity in a concentration-dependent manner, and *SQD2.2*-OE plants exhibited reduced SS activity accompanied by a significant reduction in starch levels and an elevation in soluble sugar levels relative to WT plants. Both adenosine diphosphate-glucose (ADPG) and UDPG levels in *SQD2.2*-OE plants were notably lower than those in WT plants. Taken together, rice SQD2.2 exhibits a novel role in flavonoid synthesis and plays an important role in mediating sugar allocation between primary and secondary metabolism in rice.

## Introduction

Cereal grain, mostly in the form of starch, is a major product that supplies energy for human activity, feedstock and industrial materials. The seed setting rate is a major factor controlling seed production and is a complicated trait regulated by the combination of genetic, physiological and environmental factors. Seed setting relies on source and sink allocation and is contributed to largely by starch accumulation, which is the result of complex enzymatic processes, with ADP-glucosepyrophosphorylase (AGPase) and starch synthase (SS) being key players^1–3^. Starch synthesis is catalyzed by SS through the transfer of the glucose moiety from ADPG to an elongated glucan chain in an α-1,4-linked manner. AGPase, a heterotetrameric enzyme consisting of two small subunits and two large subunits, catalyzes a reaction between glucose-1-phosphate and ATP to produce ADPG, which provides a critical substrate fueling starch synthesis^1, 4^. A deficiency of starch synthesis results in a reduced seed yield in plants^5, 6^. Increased AGPase activity in the endosperm promotes grain filling and increased seed yield in wheat and rice^2, 7^. Furthermore, starch also serves as a storage sugar and is important for plant growth. Depleting starch from leaves causes growth inhibition and delayed flowering in Arabidopsis^8^. These results suggest that starch synthesis in both the sink and the source is important for seed setting and plant growth.

Glycosylation, the process of transferring an active sugar moiety to acceptor molecules, occurs in diverse reactions involved in primary and secondary metabolic processes such as starch synthesis, flavonoid modification and galactolipid synthesis^9^. Flavonoids belong to a group of secondary metabolites composed largely of 900 molecular species, including flavonols, flavones, anthocyanins and proanthocyanidins. Glycosylation is an important process enabling the solubility and stability of hydrophobic flavonoids, and most flavonoids are present in plant tissues in glycosidic form^9, 10^. For example, a deficiency in UDP-sugar: 3-O-glycosyltransferase (UF3GT) activity results in a significant reduction in anthocyanin accumulation in Arabidopsis^11^. Glycosylation is catalyzed by UDP-sugar: glycosyltransferase (UGT), in which the C-terminus contains a conserved plant secondary product glycosyltransferase (PSPG) motif responsible for binding to the UDP moiety of sugar donors^12, 13^. The genes involved in flavonoid backbone biosynthesis have been extensively characterized in Arabidopsis. In comparison, the UGTs encoded by diverse genes belong to the glycosyltransferase (GT) super-family, which makes it difficult to screen target UGT genes. To date, more than 1500 putative UGT genes have been identified from various plant genomes based on the highly conserved PSPG motif. Many UGTs behave in a more regiospecific manner than a substrate-specific manner^9, 14^. Glycosylation usually occurs with 3-, 5- or 7-hydroxyl regiospecificity of the flavonoid core structure and is catalyzed by 3-O-glycosyltransferase (F3GT), 5-O-glycosyltransferase (F5GT) and 7-O-glycosyltransferase (F7GT), respectively^14, 15^.

Flavonoids function as pigments, ultraviolet (UV) protectants, attractants of pollinators, phytoalexins, signaling molecules, regulators of fertility and regulators of auxin transport^16–20^. The precise relationships between flavonoid species and their physiological functions are still largely unknown due to the huge diversity of structures and distribution in plants. Glycosidic apigenin (4,5,7-trihydroxyflavone), one of the flavonoids widely found in plants, functions as an antioxidant to relieve lipid peroxidation and free radicals and aid in defense^21^. Flavonoids function as multidrug-resistant proteins^20^, which are important for organismal growth, development and morphogenic modeling in response to nutrient availability and stimuli^16, 19, 22^. In addition, flavonoids are also involved in auxin transport and the jasmonic acid (JA) response^20, 22, 23^. Quercetin inhibits auxin efflux transport and multidrug-resistant proteins^20^. Pharmacological treatment showed that flavonoids inhibit the activity of various kinases involved in growth and development in eukaryotic cells^24^. The data suggest that flavonoids have multiple effects, integrating plant growth, development and the stress response. Flavonoid accumulation was found in plant tissues under different stress conditions such as salt, drought, low temperature, nutrient deficiency and UV irradiation, along with growth arrest and seed yield reduction in plants^25–28^. However, the direct link between flavonoid and sugar metabolism or seed yield is unclear. Moreover, sugars provide the substrates for flavonoid synthesis, and the crosstalk between primary metabolic functions such as starch synthesis and secondary metabolic functions remains to be elucidated in plants.

Sulfoquinovosyldiacylglycerol (SQDG) synthase (SQD) is also a glycosyltransferase that catalyzes the transfer of the sulfoquinovose moiety to diacylglycerol (DAG) to generate SQDG^29^. SQDG is a sulfur-containing anionic glycerolipid that is exclusively located in thylakoid membranes. The photosystem II complex possesses three molecules of SQDG in addition to monogalactosyldiacylglycerol (MGDG), digalactosyldiacylglycerol (DGDG) and phosphatidylglycerol (PG)^30, 31^. SQDG was also found in the cytochrome b6-f complex^30^. SQDG has a negative charge similar to PG and is capable of replacing PG under phosphate starvation conditions. In plants, SQDG is synthesized via a three-step reaction in chloroplasts. First, UDPG is synthesized from glucose-1-phosphate and uridine-5′-triphosphate (UTP) by UGP3, a plastid UDPG pyrophosphorylase (UDPase)^32^. Subsequently, SQD1, the SqdB ortholog, catalyzes a reaction between UDPG and sulfite to produce UDP-sulfoquinovose^33^. Finally, SQDG is produced by SQDG synthase, designated as SQD2, using UDP-sulfoquinovose and DAG as substrates^29^. Both UGP3 and SQD1 are soluble enzymes and are localized to the chloroplast stroma in Arabidopsis^32, 33^. SQD2 is localized to the inner envelope membrane of chloroplasts in Arabidopsis^29^. Genetic mutation in any of these enzymes, UGP3, SQD1 or SQD2, results in a complete loss of SQDG from cells, and Arabidopsis plants with these mutations exhibit growth retardation under phosphate starvation conditions^29, 32, 33^. Among the three SQDG-deficient mutants of Arabidopsis, the severe growth defect and lack of the novel anionic glycolipid glucuronosyldiacylglycerol (GlcADG) were found only in the *sqd2* mutants under phosphate-limited conditions^34^, suggesting that the function of Arabidopsis SQD2 (AtSQD2) is not only required for SQDG synthesis but also for GlcADG synthesis in Arabidopsis. These results indicate that the SQD2 enzyme may have diverse substrates in plants. In this study, we identified a SQD2-like enzyme, designated as SQD2.2, in rice (*Oryza sativa* L). Rice SQD2.2 was found to be a UGT enzyme that catalyzes the glycosylation of apigenin to produce apigenin 7-O-glucoside but has no detectable SQDG synthase activity. Overexpression of rice *SQD2.2* enhances flavonoid accumulation, resulting in a reduced seed setting rate and diminished tiller number accompanied by a reduced starch content in both source and sink tissues. Apigenin 7-O-glucoside, the product of SQD2.2 activity, inhibited SS activity. Moreover, *SQD2.2*-overexpressing (OE) plants had a significant reduction in ADPG and UDPG compared with wild-type (WT) plants, suggesting that enhanced production of flavonoid by SQD2.2 results in sugar substrate competition and thus a reduced requirement for ADPG for starch synthesis. These results suggest that rice SQD2 exhibits multifaceted enzymatic properties with different substrate selections and regulates the partitioning of carbon between primary and secondary metabolism in rice.

## Results

### Identification of the SQDG Synthase in Rice

SQD2 belongs to the GT superfamily that contains a glycosyltransferase catalytic domain (Fig. S1). To functionally characterize the role of rice SQD2, homology searches and protein domain structure prediction were performed using AtSQD2 as a query, and three putative *SQD2* genes were identified from the rice genome and were designated as *SQD2.1, SQD2.2* and *SQD2.3* based on sequence similarity (Fig. S1). Rice SQD2.1, SQD2.2 and SQD2.3 share 72%, 67% and 68% amino acid sequence similarity to Arabidopsis SQD2, respectively. Rice SQD2.1 is related most closely to Arabidopsis SQD2. Phylogenetic analysis showed that SQD2.2 is classified into the group of SQD2 homologs in Arabidopsis and *Synechococcus elongatus* PCC7924 but is relatively distant from the UGTs involved in flavonoid glycosylation (Fig. S2). Rice *SQD2.2* encodes a protein of 515 amino acids with a predicted pI of 7.23 and a molecular weight of 57.9 kDa. The role of SQD2.2 is unknown.

### Overexpression of *SQD2.2* Results in Reduced Seed Setting

**Figure 1:**
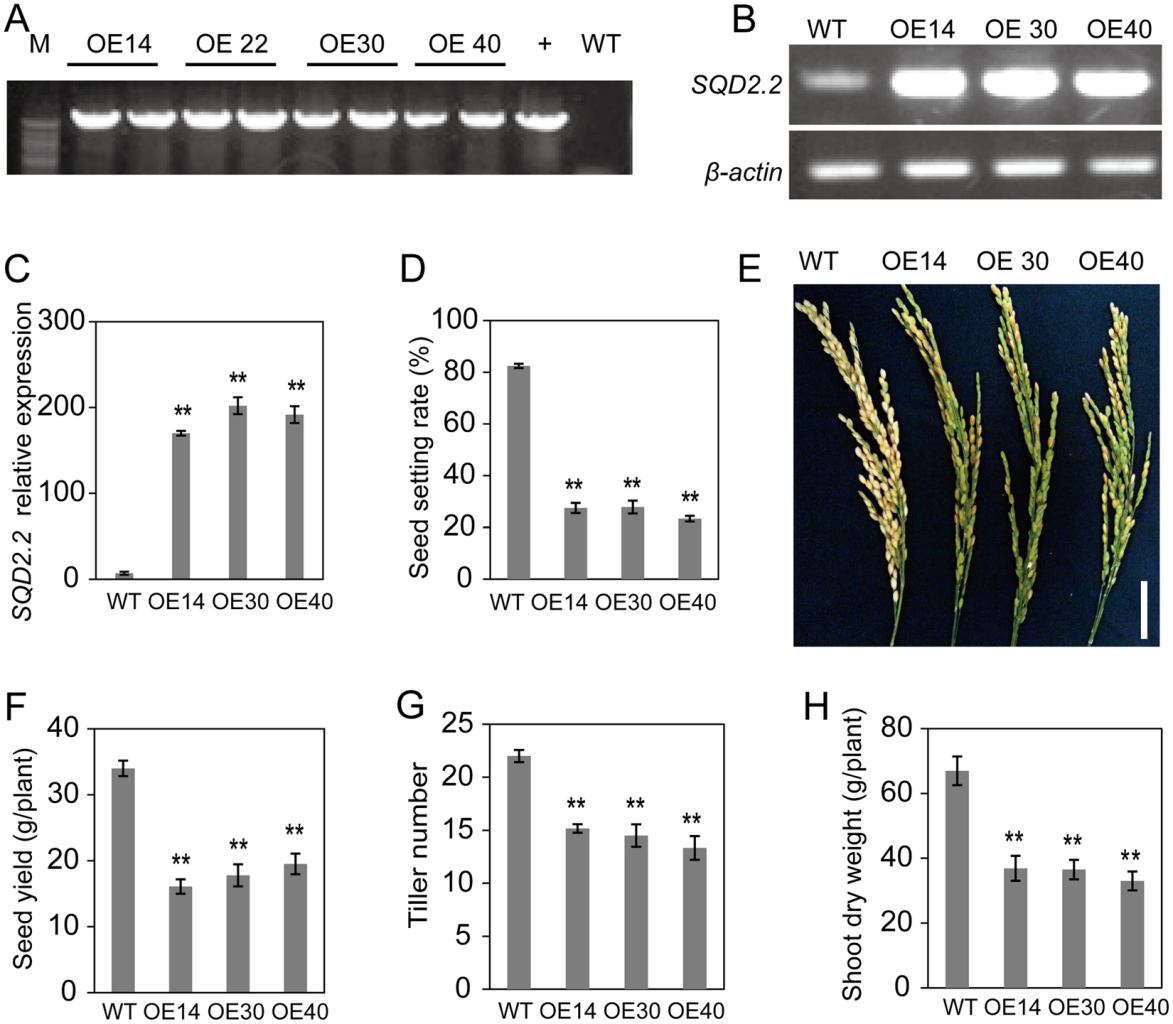
Overexpression of *SQD2.2* resulted in a reduced seed setting rate and tiller number. (**A**) Identification of transgenic plants using PCR. (**B**) and (**C**) *SQD2.2* overexpression in the transgenic plants was confirmed via semi-quantitative RT-PCR and quantitative real-time PCR. Values are the mean ± standard error (SE) (n = 3). (**D**) and (**E**) Seed setting rate. Values are the mean ± SE (n = 10). (**F**) Seed yield per plant. Values are the mean ± SE (n = 10). (**G**) The tiller number per plant. Values are the mean ± SE (n = 6). (**H**) Shoot dry weight per plant. Values are the mean ± SE (n = 10). Student’s *t* test; **P < 0.01. Scale bar = 5 cm.

To explore the role of rice *SQD2.2* encoding a putative SQDG synthase, a full-length cDNA of rice *SQD2.2* was cloned and overexpressed in rice under the control of the maize *Ubiquitin* promoter. A total of 41 transgenic lines were obtained and were confirmed by PCR. The sequencing result of PCR product in Fig. 1A is identical to the sequence of *SQD2.2* cDNA from data base (Fig. S3). Most transgenic lines exhibited a significantly higher transcript level than that of WT plants without transformation. Overexpression of *SQD2.2* resulted in a decreased seed setting rate under normal growth conditions in comparison to WT. To further explore the role of SQD2.2, three OE lines, namely, OE14, OE30 and OE40, were randomly selected for detailed characterization (Fig. 1A–C). The seed setting rates of OE14, OE30 and OE40 plants were 27.5%, 27.8% and 23.3%, respectively, whereas that of WT was 82.4% (Fig. 1D and E). The seed yields in OE14, OE30 and OE40 were 47.3%, 52.3% and 57.3% of that in WT plants (Fig. 1F). In addition, the overexpression of *SQD2.2* also led to reduced total tiller number and shoot biomass (Fig. 1G and H). The pollen viability in OE plants was not significantly different from that in WT plants (Fig. S4), suggesting that the reduced seed setting rate did not result from pollen fertility.

### *SQD2.2* Overexpression Enhances Flavonoid Accumulation

**Figure 2:**
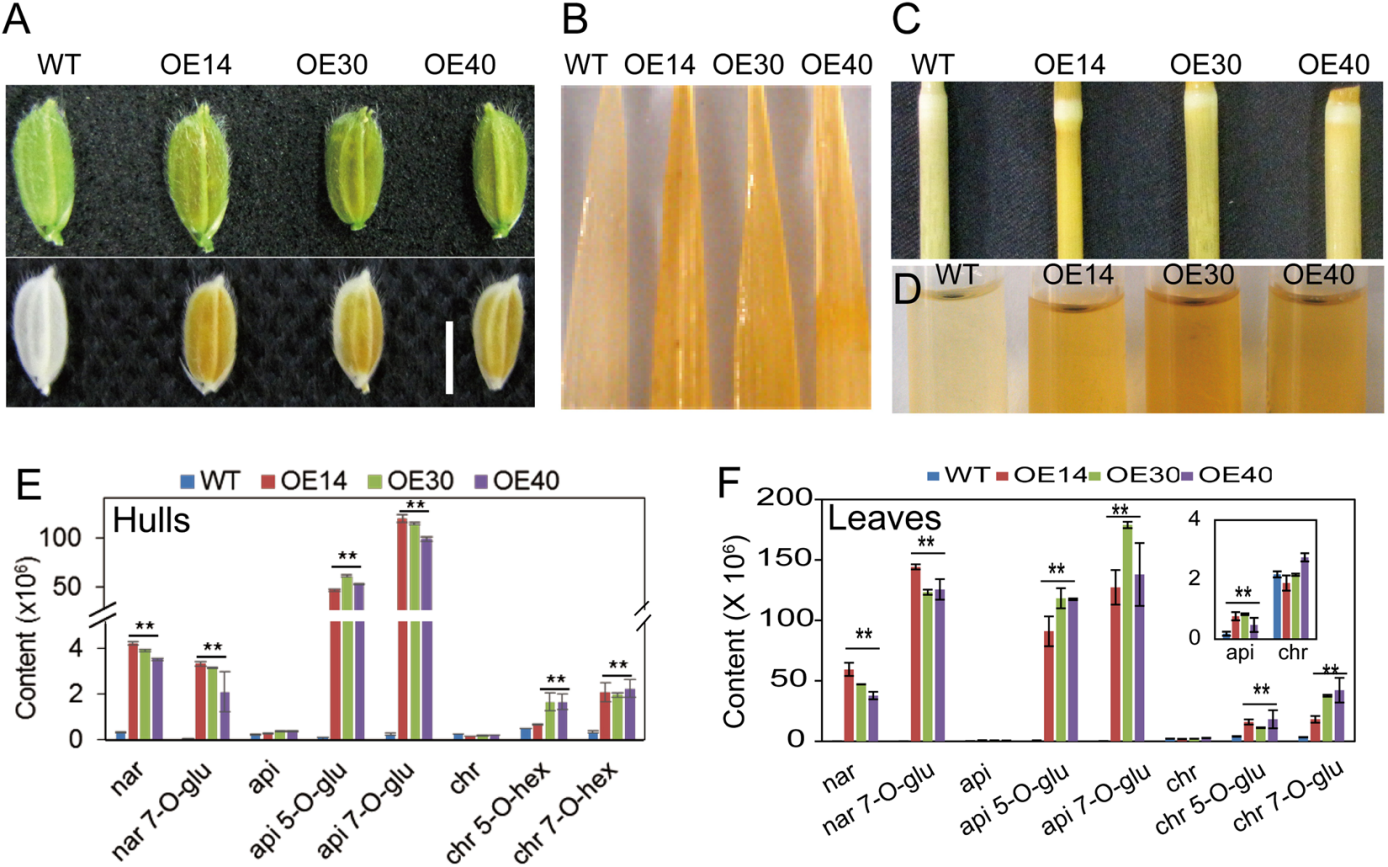
Overexpression of *SQD2.2* enhanced flavonoid accumulation in various tissues. (**A**) to (**D**) Flavonoid in hulls (**A**), leaves (**B**) and stems (**C**) and methanol extracts from leaves (**D**). Plants were grown in a paddy field under normal growth conditions, and the samples were photographed after chlorophyll was removed by ethanol. (**E**) and (**F**) Overexpression of *SQD2.2* increased flavonoid levels, with the predominant accumulation of 7-O-glycosidic and 5-O-glycosidic flavonoids in seed hulls (**E**) and leaves (**F**). nar, naringenin; nar 7-O-glu, naringenin 7-O-glucoside; api, apigenin; api 5-O-glu, apigenin 5-O-glucoside; api 7-O-glu, apigenin 7-O-glucoside; chr, chrysoeriol; chr 5-O-hex, chrysoeriol 5-O-hexoside; chr 7-O-hex, chrysoeriol 7-O-hexoside. Values are the mean ± SD (n = 6). Student’s *t* test; **P < 0.01. Scale bar = 0.5 cm.

In addition to the reduced seed setting and tiller number, the overexpression of *SQD2.2* also conferred plants with enhanced flavonoid levels in various tissues, including hulls, leaves and stems (Fig. 2A–D). Brown pigments were observed in *SQD2.2-*OE tissues and were much more obvious than those in WT plants after the chlorophyll was removed from the tissues (Fig. 2A–D). To determine the flavonoid molecular species, the brown pigments were extracted from the hulls and leaves of *SQD2.2-*OE and WT plants using methanol, and the extracts were subjected to liquid chromatography electrospray ionization–tandem mass spectrometry (LC ESI–MS/MS) profiling. The overexpression of *SQD2.2* led to a dramatic increase in glycosidic flavonoids, including apigenin 5-O-glucoside, apigenin 7-O-glucoside, naringenin 7-O-glucoside, chrysoeriol 5-O-hexoside and chrysoeriol 7-O-hexoside; in particular, apigenin 7-O-glucoside and apigenin 5-O-glucoside were greatly increased, with 1,000-fold and 500-fold increases in OE plants relative to WT plants, respectively (Fig. 2E and F). By comparison, the flavonoids without glycosylation, such as apigenin and chrysoeriol, exhibited a small increase in *SQD2.2-*OE plants, and naringenin was also elevated to some extent relative to WT (Fig. 2E and F). These results suggest that the overexpression of *SQD2.2* enhances flavonoid accumulation, with a predominant increase in glycosidic flavonoid derivatives in rice. Moreover, the overexpression of rice *SQD2.2* in Arabidopsis also led to flavonoid, especially anthocyanin, accumulation in various tissues, including leaves, stems and siliques (Fig. S5), suggesting that SQD2.2 is able to enhance flavonoid accumulation in both monocot and dicot plants.

### *SQD2.2* Encodes a Flavonoid Glycosyltransferase

**Figure 3:**
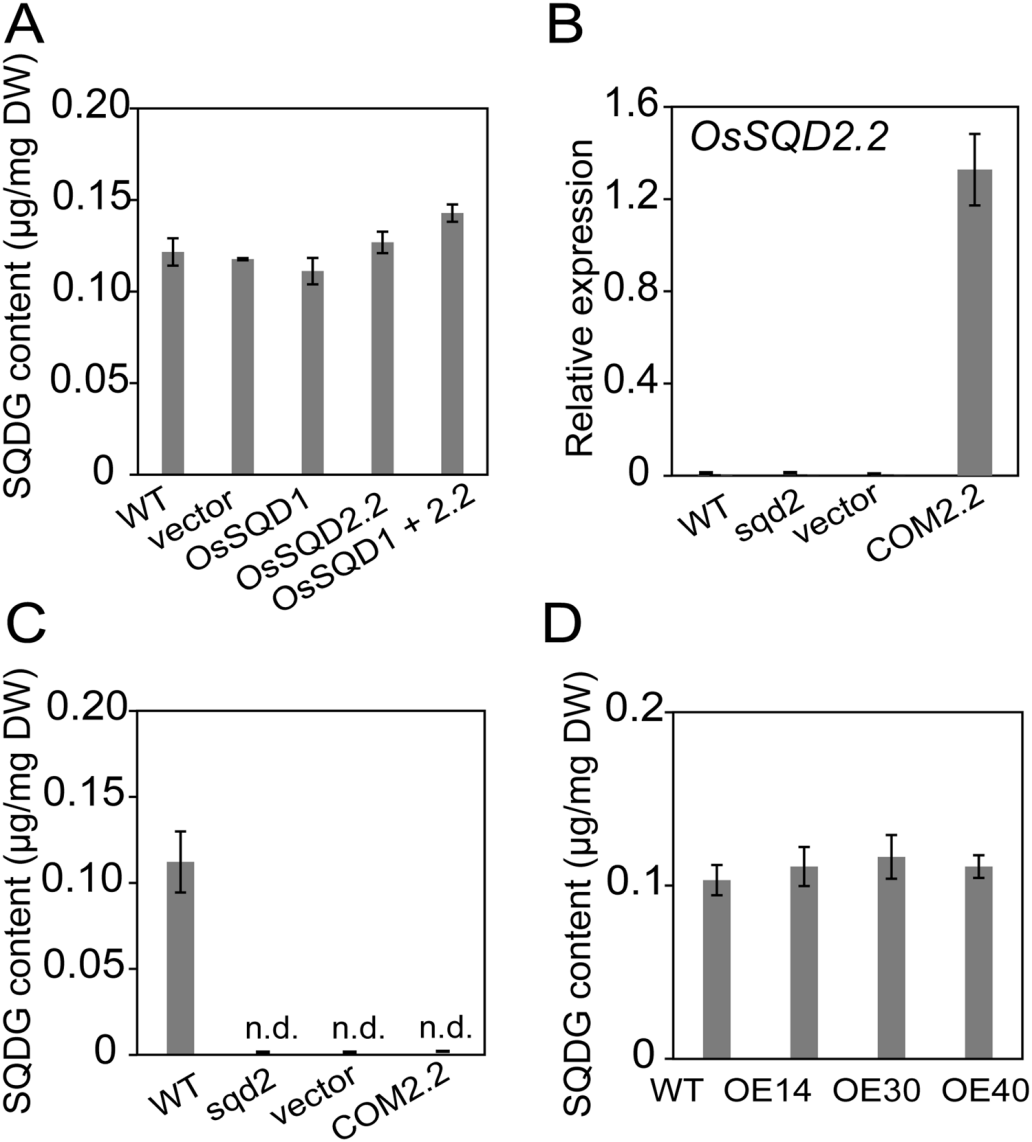
SQD2.2 has no detectable SQDG synthase activity. (**A**) Co-expression of *SQD1* and *SQD2.2* in tobacco leaves did not result in elevated SQDG content relative to the controls, WT, vector and leaves transformed with either *SQD1* or *SQD2.2* individually. Values are the mean ± SE (n = 3). (**B**) and (**C**) Genetic complementation with rice *SQD2.2* in the Arabidopsis *sqd2* mutant failed to restore SQDG synthesis. Lipids were extracted from the leaves of four-week-old seedlings and separated by TLC for GC measurement. Values are the mean ± SE (n = 3). COM2.1, COM2.1, the plants that were genetically complemented with rice *SQD2.1* and *SQD2.2*, respectively, in the Arabidopsis At*sqd2* mutant. n.d., not detectable. (**D**) Overexpression of *SQD2.2* in rice plants did not increase SQDG content relative to WT plants. Lipids were extracted from leaves at the seed filling stage. Values are the mean ± SE (n = 3).

In Arabidopsis, SQDG synthase, designated SQD2, catalyzes the final reaction step in SQDG synthesis by transferring the sulfoquinovose group to DAG^29^. Rice SQD2.2 also contains a conserved glycosyltransferase catalytic domain and shares 67% sequence similarity with Arabidopsis SQD2 (Fig. S1). To examine whether rice *SQD2.2* encodes an SQDG synthase, *SQD2.2* was transiently coexpressed with rice *SQD1*, the gene encoding an enzyme for UDP-sulfoquinovose synthesis, in tobacco leaves. The resultant lipids were extracted and quantitatively analyzed using GC. The SQDG content in the leaves of *SQD1*/*SQD2.2* co-expressing plants was not significantly different from that in the controls, including WT plants, plants transformed with the vector only or plants transformed with either *SQD1* or *SQD2.2* individually (Fig. 3A). These results suggest that rice SQD2.2 had no detectable SQDG synthase activity under the condition tested. To further confirm this result, rice *SQD2.2* was ectopically expressed in an Arabidopsis *sqd2* mutant (Fig. 3B). The Arabidopsis *sqd2* mutant exhibited defective SQDG, and the ectopic expression of rice *SQD2.2* in the mutant failed to restore SQDG synthesis (Fig. 3C). In addition, the overexpression of *SQD2.2* did not confer an increased SQDG level in the plants in comparison to WT (Fig. 3D). Taken together, the results indicate that rice SQD2.2 does not exhibit SQDG synthase activity.

**Figure 4:**
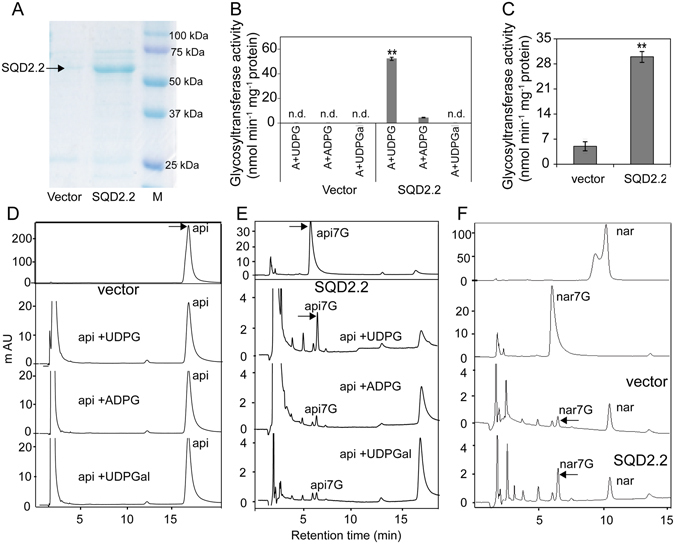
SQD2.2 catalyzed the transfer of glucose from UDPG to apigenin and naringenin to generate apigenin 7-O-glucoside and naringenin 7-O-glucoside. (**A**) SQD2.2 protein was expressed in *E. coli* cells and was purified for the enzymatic activity assay. (**B**), (**D**) and (**E**) SQD2.2 activity toward apigenin to produce apigenin 7-O-glucoside using UDPG, but not ADPG or UDPGal, as a sugar donor (**B**,**E**). Protein from the cells expressing empty vector only was used as a control (**D**). Values are the mean ± SE (n = 3). (**C**) and (**F**) SQD2.2 activity toward naringenin to produce naringenin 7-O-glucoside using UDPG as a sugar donor. Purified SQD2.2 protein (5 μg) was added to 100 μl of the reaction mixture. Protein from the cells expressing empty vector only was used as a control. n.d., not detectable; M, protein ladder marker; A (api), apigenin; api7G, apigenin 7-O-glucoside; nar, naringenin; nar7G, naringenin 7-O-glucoside; UDPG, UDP-glucose; ADPG, ADP-glucose; UDPGal, UDP-galactose. Values are the mean ± SE (n = 3). Student’s *t* test; **P < 0.01.

The overexpression of *SQD2.2* resulted in a significant increase in flavonoids, with a dramatic elevation in glycosidic flavonoids (Fig. 2), but it had no effect on SQDG content relative to WT plants (Fig. 3D), implicating that rice *SQD2.2* may encode a flavonoid glycosyltransferase rather than a SQDG synthase. To test this possibility, full-length rice *SQD2.2* cDNA was cloned into the pET28a vector to express the protein. The recombinant SQD2.2-His protein, which has a molecular weight of 57.9 kDa, was expressed in *E. coli* cells and was purified for an enzymatic activity assay (Fig. 4A). The *in vitro* assay indicated that SQD2.2 catalyzes the transfer of the glucose moiety from UDPG to 7-hydroxyl of apigenin to produce apigenin 7-O-glucoside (Fig. 4B and E). In comparison, the control cells containing empty vector did not exhibit detectable levels of apigenin 7-O-glucoside (Fig. 4D). To further test the substrate preference, different sugar donors were used for the enzymatic activity assay. The results showed that SQD2.2 catalyzed the transfer of the glucose moiety from UDPG to apigenin but failed to use ADPG and UDP-galactose (UDPGal) as sugar donors for apigenin (Fig. 4B and E). In addition, SQD2.2 was also able to transfer the glucose moiety of UDPG to naringenin to produce naringenin 7-O-glucoside (Fig. 4C and F). These results demonstrate that rice *SQD2.2* encodes a flavonoid glycosyltransferase rather than an SQDG synthase. SQD2.2 is directly responsible for flavonoid accumulation, but not for SQDG synthesis, in rice plants.

### *SQD2.2* is Predominantly Expressed in Green Tissues, and SQD2.2 is Predominantly Localized to the Cytoplasm

**Figure 5:**
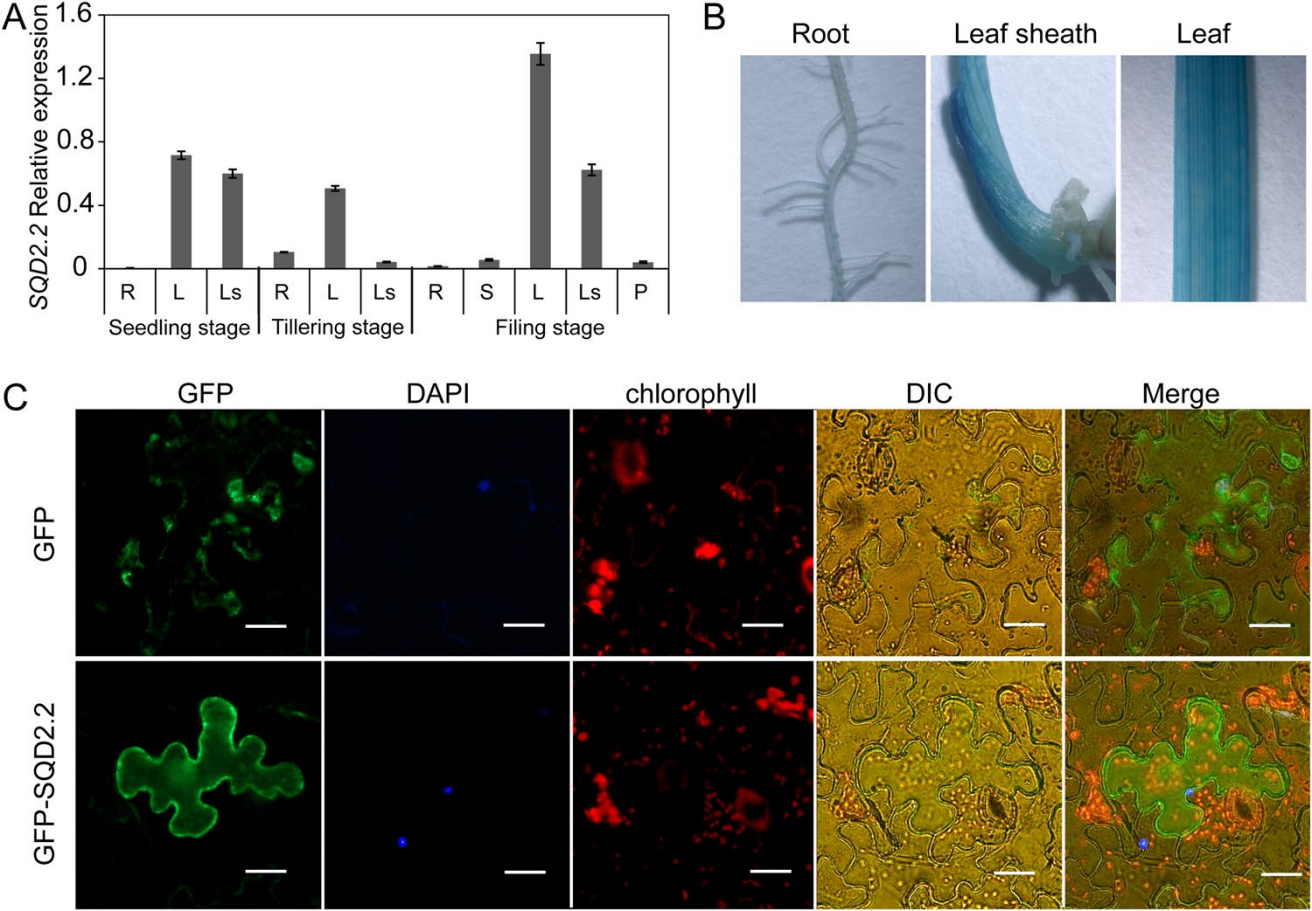
Expression patterns of *SQD2.2* in rice and SQD2.2 is localized to the cytoplasm. (**A**) *SQD2.2* is expressed in different tissues. The *SQD2.2* transcript level was quantified with real-time PCR using *β-actin* as an internal standard. R, roots; S, stems; L, leaves; Ls, leaf sheaths; P, panicles. Values are the mean ± SE (n = 3). (B) Histochemical GUS staining patterns of *pSQD2.2*::GUS transgenic plants. (**C**) GFP-SQD2.2 was transiently expressed in tobacco epidermal cells, and the subcellular localization was visualized under a fluorescence microscope. Scale bar = 10 μm.

Quantitative real-time PCR revealed that *SQD2.2* is expressed in various organs, including leaves, roots, stems and panicles, with higher levels in leaves and lower levels in roots at the different stages tested; *SQD2.2* levels were highest in leaf blades during the mature stage (Fig. 5A). The *SQD2.2* transcript level in leaf sheaths was also relatively high during the seedling and mature stages (Fig. 5A). It exhibited a similar pattern by histochemical GUS staining in rice plants containing GUS gene under the control of *SQD2.2* promoter (Fig. 5B). To test its subcellular localization, SQD2.2 was fused with GFP and was transiently expressed in the epidermal cells of tobacco leaves. The GFP-SQD2.2 fusion protein was predominantly localized in the cytoplasm (Fig. 5C), which differs from the chloroplast-localized AtSQD2^29^. The cytosolic localization of rice SQD2.2 further demonstrates its role in flavonoid glycosyltransferase rather than SQDG synthesis, which occurs exclusively in chloroplasts in plants.

### Starch Synthase (SS) Activity is Inhibited by Glycosidic Flavonoids

**Figure 6:**
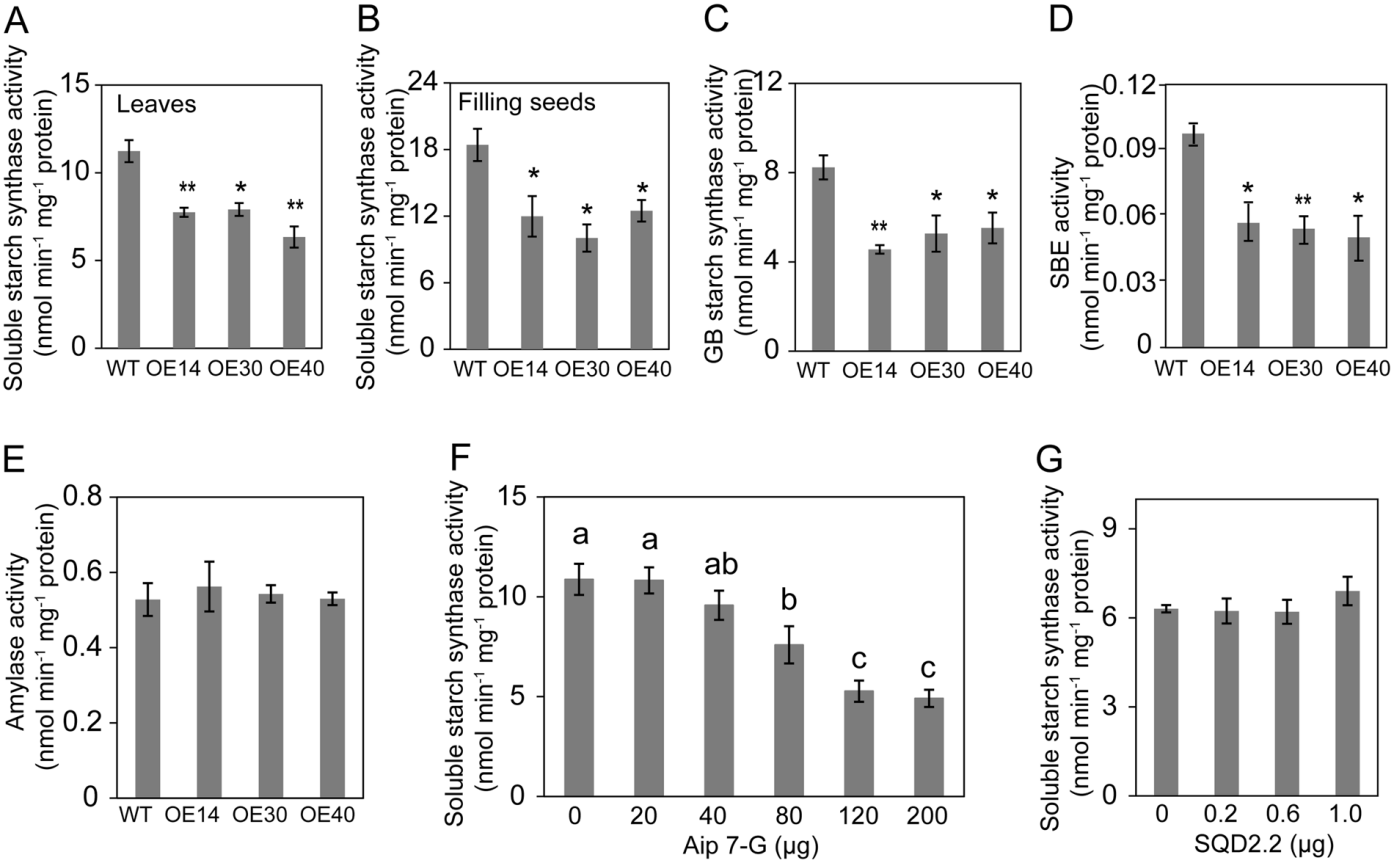
Apigenin 7-O-glucoside inhibited starch synthase activity, and overexpression of *SQD2.2* resulted in reduced starch synthase activity. (**A**) and (**B**) Soluble starch synthase activity in the leaves and filling seeds of *SQD2.2*-OE was significantly lower than in that in WT plants. Proteins were extracted from leaves at the tillering stage and extracted from the filling seeds 15 days after anthesis. The same amount of protein (100 μg) from both *SQD2.2*-OE and WT plants was added to the reaction mixture. Values are the mean ± SE (n = 6). (**C**) to (**E**)The activities of granule-bound (GB) starch synthase (**C**), starch branching enzyme (SBE) (**D**) and amylase (**E**) in leaves of *SQD2.2*-OE and WT plants. Proteins were extracted from leaves at the tillering stage. Values are the mean ± SE (n = 6). (**F)** Apigenin 7-O-glucoside, the product of SQD2.2, inhibited soluble starch synthase activity in a concentration-dependent manner. Proteins were extracted from the leaves of one-month-old WT plants. Values are the mean ± SE (n = 3). Different letters (a, b and c) indicate significant differences at P < 0.05, according to ANOVA analysis and Duncan’s multiple range test. Api 7-G, apigenin 7-O-glucoside. (**G**) SQD2.2 itself did not inhibit soluble starch synthase activity. Proteins were extracted from the leaves of one-month-old WT plants and were treated with purified SQD2.2 protein in various concentrations. Values are the mean ± SE (n = 3). Student’s *t* test; *P < 0.05, **P < 0.01.

Overexpression of *SQD2.2* led to increased flavonoid levels in various tissues accompanied by a reduced seed setting rate. To test the effect of SQD2.2 on starch synthase activity *in vivo*, the different classes of enzymes contributing to starch synthesis were measured at the tillering and seed filling stages. The soluble SS activity in the leaves of *SQD2.2-*OE plants was significantly lower than that in WT and was decreased by 31.1%, 29.6% and 43.6% in the leaves of OE14, OE30 and OE40, respectively (Fig. 6A). Likewise, the soluble SS activity in the filling seeds of *SQD2.2-*OE plants was significantly decreased at 15 days after anthesis, with 35%, 45.5% and 32.2% reductions in the filling seeds of OE14, OE30 and OE40, respectively, relative to WT (Fig. 6B). In addition, the activities of granule-bound SS (GBSS) and starch branching enzyme (SBE) were reduced in OE plants (Fig. 6C and D). The activity of amylase, the enzyme involved in starch degradation, did not differ between OE and WT plants (Fig. 6E). To test whether glycosidic flavonoids directly affect starch biosynthesis, SS activity was monitored in response to apigenin 7-O-glucoside treatment at various concentrations. The results showed that SS activity was significantly reduced in the presence of apigenin 7-O-glucoside and that the inhibition occurred in a concentration-dependent manner (Fig. 6F). In comparison, the SQD2.2 protein itself did not significantly influence SS activity (Fig. 6G).These data suggest that SQD2.2 catalyzes the synthesis of glycoside flavonoids, which inhibits starch synthesis and thus reduces seed setting.

### SQD2.2 Affects Sugar Allocation between Primary and Secondary Metabolism

**Figure 7:**
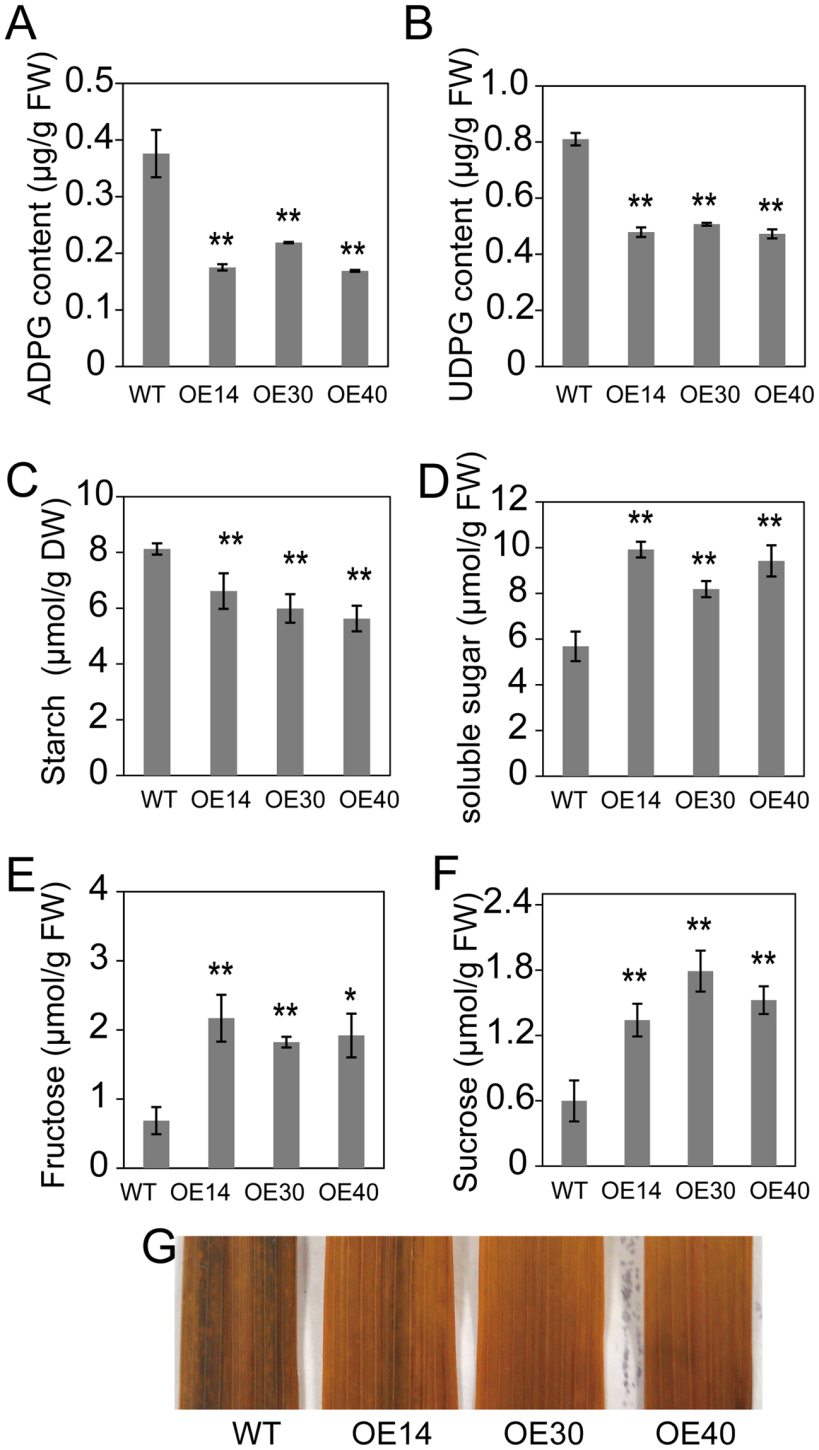
Overexpression of *SQD2.2* resulted in reduced UDPG and ADPG content and decreased starch synthesis. (**A**) and (**B**) The ADPG and UDPG content in *SQD2.2*-OE leaves was significantly lower than that in WT plants at the tillering stage. Values are the mean ± SE (n = 6). (**C**) to (**F**) Reduced starch and elevated soluble sugar levels in *SQD2.2*-OE plants compared to WT plants. Samples were taken at the tillering stage. Values are the mean ± SE (n = 6). (**G**) Starch accumulation in leaves was visualized by KI-I_2_ staining. Leaf pigments were removed by 96% ethanol, and then leaves were stained with iodine solution (2% KI, 1% I_2_) for 30 min. Student’s t test; *P < 0.05, **P < 0.01.

SQD2.2 used UDPG to participate in the synthesis of glycosidic flavonoids (Fig. 4), while ADPG is a critical substrate for starch biosynthesis^35^. Both ADPG and UDPG share a common substrate, glucose-1-phosphate. To investigate whether the overexpression of *SQD2.2* affects ADPG synthesis due to sugar substrate competition, ADPG and UDPG levels were measured in *SQD2.2*-OE and WT plants. The ADPG contents in all three *SQD2.2*-OE lines were significantly lower than that in WT plants, demonstrating decreases of 53.5%, 41.8% and 55.1% in OE14, OE30 and OE40 plants, respectively (Fig. 7A). Likewise, UDPG also exhibited a similar tendency (Fig. 7B). These results suggest that upregulated SQD2.2 in OE plants leads to increased consumption of UDPG, thus significantly reducing the ADPG available for starch synthesis due to substrate competition.

To investigate whether overexpression of *SQD2.2* influences sugar metabolism, the starch and soluble sugar content were tested in the leaves of *SQD2.2-*OE and WT plants on a sunny day during the heading stage. The starch content in *SQD2.2-*OE leaves was significantly lower than that in WT plants; the starch content in OE14, OE30 and OE40 was 81.4%, 73.7% and 69.3% of that in WT, respectively (Fig. 7C and G). In contrast, the level of soluble sugars, including fructose and sucrose, in the leaves of *SQD2.2-*OE plants exhibited a two-fold increase over the level in WT plants (Fig. 7D–F). These results suggest that overexpression of *SQD2.2* led to reduced starch accumulation and an increase in soluble sugars. Taken together, these data suggest that SQD2.2 catalyzes the synthesis of glycosidic flavonoids, which inhibits SS activity and reduces the carbon flux for starch synthesis, thus reducing the seed setting rate and biomass accumulation in rice.

## Discussion

### Rice SQD2.2 is Involved in Flavonoid Glycosylation Rather than SQDG Synthesis

SQDG belongs to a class of sulfur-containing lipids that are primarily located in the thylakoid membrane and is conditionally important for photosynthetic bacteria, alga and plants due to its ability to replace anionic PG in response to phosphate limitation. Loss of SQDG results in defective growth under phosphate-starvation conditions^29, 34, 36, 37^. In Arabidopsis and spinach, SQDG is exclusively synthesized in chloroplasts, as demonstrated by the chloroplast localization of all enzymes involved in SQDG synthesis^29, 32–34^. The Arabidopsis genome contains one gene, *SQD2*, which encodes an enzyme involved in the final step of the SQDG synthesis pathway^29^. To date, none of the SQD2 orthologs has been functionally characterized in rice. Through homology searches, we found that the rice genome contains three homologous genes, designated *SQD2.1*, *SQD2.2* and *SQD2.3*. Their encoding proteins﻿ share high sequence similarity and contain a conserved glycosyltransferase domain (Fig. S1), suggesting that rice SQD2 orthologs may be more complicated and functionally diverse. Indeed, the data from this study showed that rice SQD2.2 is predominantly localized to the cytoplasm and failed to restore Arabidopsis *sqd2* mutant to a WT phenotype, whereas rice SQD2.1 is localized to chloroplasts and is capable of complementing SQDG synthesis in Arabidopsis *sqd2* mutant plants. Rice SQD2.2 shares 67% sequence similarity with AtSQD2. Unexpectedly, rice SQD2.2 was identified to be a glycosyltransferase that acts on flavonoids such as apigenin and naringenin to produce glycosidic flavonoids. Furthermore, overexpression of *SQD2.2* had no influence on the SQDG content but caused a notable increase in glycosidic flavonoids (Figs 2 and 4). These data suggest that SQD2.2 is primarily involved in flavonoid accumulation rather than in SQDG synthesis in rice. SQD2 orthologs possess a catalytic domain involved in transferring a sugar moiety to an acceptor, usually DAG, to produce SQDG^29^. However, a recent study showed that ﻿Arabidopsis ﻿SQD2 is also involved in GlcADG synthesis, but its precise substrate is unclear^34^. The current results show that rice SQD2.2, an enzyme homologous to AtSQD2, catalyzes the conversion of apigenin and naringenin to apigenin 7-O-glucoside and naringenin 7-O-glucoside, respectively. These results suggest that plant SQD2 orthologs are capable of using different substrates, including DAG and flavonoids, as sugar moiety acceptors and are involved in diverse metabolic processes, such as sulfolipid biosynthesis and flavonoid glycosylation, in plants.

### SQD2.2 Exerts Dual Effects on Starch Synthesis to Mediate Primary and Secondary Metabolic Processes

Cereal grain is mostly composed of starch in the endosperm, and starch synthesis contributes largely to seed setting. Increased AGPase activity resulted in enhanced starch accumulation and increased seed yield in rice and wheat^1, 2^. Reduced seed setting occurred simultaneously with glycosidic flavonoid accumulation in *SQD2.2*-OE plants (Figs 1 and 2), suggesting that the glycosidic flavonoids produced by SQD2.2 have a negative effect on seed setting. Glycosylation facilitates the solubility and stability of hydrophobic flavonoids^10, 12^. Flavonoids are involved in various biological processes such as stress response and hormone signaling^16, 23, 28, 38^. Whether flavonoids affect starch synthesis remains unknown. An enzymatic assay showed that SQD2.2 directly regulates glycosidic flavonoid accumulation. Moreover, increased flavonoid levels were accompanied by reduced starch content in both the leaves and filling seeds of *SQD2.2*-OE plants, suggesting that the glycosidic flavonoids produced by SQD2.2 have a negative effect on starch synthesis. Indeed, the SS activity was specifically inhibited by apigenin 7-O-glucoside in a concentration-dependent manner but not by SQD2.2 protein. Furthermore, *SQD2.2*-OE plants exhibited decreased different classes of SS activity in both leaves and filling seeds relative to WT plants (Fig. 6). The reduced starch and seed setting could be explained, at least in part, by an inhibitory effect of glycosidic flavonoids on SS activity, thus disturbing sugar carbon allocation at the sink and source. Flavonoids also inhibit the activity of kinases that control key processes involved in growth and development in animal cells^24, 39^.

Nevertheless, the negative effect of flavonoids on starch synthesis may also be due to sugar competition between the glycosidic flavonoid and starch synthesis pathways. SS catalyzes the transfer of the glucose moiety from ADPG to an elongated polymer for starch synthesis^40, 41^. The enzymatic activity assay showed that SQD2.2 selectively used UDPG as a sugar donor for glycosidic flavonoid synthesis, and *SQD2.2*-OE plants accumulated a large amount of glycosidic flavonoids, with a 1,000-fold increase in apigenin 7-O-glucoside relative to WT plants (Fig. 2E and F). The results suggest that the increased levels of glycosidic flavonoids in *SQD2.2*-OE plants may consume a large amount of UDPG, thus reducing the ADPG level in starch synthesis, as both ADPG and UDPG share a common precursor, glucose-1-phosphate, and are generated by AGPase and UDPase, respectively^1^. ADPG is a critical substrate for starch synthesis. The overexpression of AGPase genes enhanced the starch content in wheat, rice and maize^1, 2, 4, 42^. The current study found that the levels of both ADPG and UDPG in *SQD2.2*-OE plants were significantly less than those in WT plants. Increasing the level of glycosidic flavonoids occurs at the expense of starch synthesis. Therefore, the reduced starch synthesis in *SQD2.2*-OE plants resulted from the dual effects of SQD2.2, the inhibitory effect of glycosidic flavonoids on SS activity and sugar substrate competition due to enhanced SQD2.2 glycosyltransferase activity toward flavonoids.

### The Effect of SQD2.2 on Tillering and Biomass

Flavonoids usually accumulate in response to osmotic stress and nutrient deficiency and are important for the adaptation of plants to ever-changing environments due to their roles in regulating ROS homeostasis and mitigating oxidative damage^19, 43^. In addition to a reduced seed setting rate, *SQD2.2*-OE plants also exhibited a decreased tiller number and thus decreased biomass compared to WT plants (Fig. 1), suggesting the negative effect of SQD2.2 on vegetative growth. Starch is an essential component required for seed filling as well as functions as an important sugar storage molecule to effectively provide nutrients and energy for plant growth^44–46^. Reduced starch and increased soluble sugar levels may be responsible for the reduced tiller number and biomass. The starch synthesis deficiency mutants *ss4* and *ss3 ss4* had a deleterious effect on growth and pale phenotypes in Arabidopsis^35, 47^. It was shown that flavonoids accumulated in response to various stimuli, such as drought, salt stress, nitrogen limitation and phosphorus deficiency, in plants^27, 48^. The overexpression of *SQD2.2* resulted in alterations in both primary and secondary metabolites, including reduced starch content, enhanced flavonoids and increased soluble sugars relative to WT plants. Thus, the reduced tiller number may be the result of two effects, the diminished starch for energy and nutrient supply and the increased flavonoid and soluble sugar levels that resemble nutrient deficiency and osmotic stress.

## Materials and Methods

### Plant Materials and Growth Conditions

Rice (*Oryza sativa*. L) plants were derived from cv Zhonghua 11 (ssp. *Japonica*). Plants were grown in soil at the experimental field of Huazhong Agriculture University (Wuhan, China) during their natural growing season. Surface-sterilized Arabidopsis seeds (Columbia and Wassilewska) were germinated on 0.8% (w/v) agar-solidified Murashige and Skoog (MS) medium supplemented with 1% (w/v) sucrose. After 10 days, the seedlings were transferred to soil in a growth chamber under conditions of 22 °C (14 h light/10 h dark), a photosynthetic photon flux density of 200–300 μmol·m^−2^·s^−1^ and 60% relative humidity.

### *SQD2.2* Cloning and Plant Transformation

Full-length rice *SQD2.2* cDNA was amplified from cDNA synthesized from the total RNA of leaves using the forward primer 5′-GGTACCATGGAATATCCCCCACAATT-3′ paired with the reverse primer 5′-GGATCCGTGTTGTGTGATTCTGTTGA-3′. The purified PCR product was inserted into the pMD18-T vector (TaKaRa). After sequencing confirmation, the fragment was released by *BamH*I and *Kpn*I digestion and was cloned into the pCAMBIA1301U vector under the control of the maize *Ubiquitin* promoter. The resultant construct was transformed into *Agrobacterium tumefaciens* EHA105, which was used to infect the rice callus (cv. Zhonghua 11) according to a protocol described previously^49^. For genetic complementation tests, the construct was also transformed into the Arabidopsis *Atsqd2* mutant via flower dipping with an *Agrobacterium* cell suspension.

### Subcellular Localization of SQD2.2

Full-length *SQD2.2* cDNA was fused with a GFP tag at the 5′-end and cloned into the pCAMBIA1301 vector after digestion with *BamH*I and *Kpn*I. The construct was introduced into *Agrobacterium tumefaciens* GV3101 and then was infiltrated into tobacco leaves (*Nicotiana benthamiana*) for transient expression under the control of the 35S promoter. After 2 to 3 days of infection, the GFP-SQD2.2 fusion protein was observed under a fluorescence microscope (Olympus, BX53) using a 488 nm excitation and a 500–530 nm emission. Chlorophyll auto-fluorescence was detected with 570 nm excitation and 640 nm emission.

### SQD2.2 Protein Expression, Purification and Activity Assay

Full-length *SQD2.2* cDNA was amplified using the forward primer 5′-GGATCCATGGAATATCCCCCACAATT-3′ and the reverse primer 5′-AAGCTTGTGTTGTGTGATTCTGTTGA-3′. The fragment was ligated into the pET28a vector after digestion with *BamH*I and *Hind*III. The construct was transformed into *E. coli* BL21 cells for protein expression. The protein was extracted and was purified using Ni-NTA resin (Sangon, Shanghai, China) according to the manufacturer’s instructions. Purified SQD2.2 was used for activity assays in 100 μl of reaction mixture containing 10 μM flavonoid (apigenin or naringenin [Sigma]), 2 mM nucleotide sugar (UDPG, ADPG, or UDPGal [Sigma]), and 50 mM Tris-HCl, pH 7.5, at 30 °C for 1 h. Reactions were stopped by the addition of 100 μl of methanol. After filtration, the reaction mixture was subjected to high-performance liquid chromatography (HPLC) (Shimadzu, SPD-M20A) equipped with C18-RP columns (5 μm, 4.6 × 150 mm) for analysis. The mixtures were separated by a linear gradient from 40% to 60% methanol containing 0.1% acetic acid with a flow rate of 1 ml min^−1^ and UV-visible detection was performed at 254 nm.

### Quantitative Real-time PCR

Total RNA was isolated from different tissues using the TransZol reagent (TransGen Biotech, Beijing). RNA extracts were treated with DNaseI (NEB, UK) to eliminate DNA contamination. First-strand cDNA was produced from the RNA template by reverse transcription using the TIANscript RT Kit according to the manufacturer’s instructions (TransGen Biotech, Beijing). Quantitative real-time PCR was performed as described previously^50^. The primers for *SQD2.2* were 5′-GCCGTGTTCACTGGAATGATGCAA-3′ (forward) and 5′-ATATCAGGAACACCACCAGCACGA-3′ (reverse). *β-actin* was used as an internal standard with primers 5′-TATGGTCAAGGCTGGGTTCG-3′ (forward) paired with 5′-CCATGCTCGATGGGGTACTT-3′ (reverse).

### Flavonoid Measurement by LC–ESI–MS/MS

Fully expanded leaves with a similar position, age and size were sampled and ground into homogenate using liquid nitrogen. After removing chlorophyll, the precipitates were suspended in 2 ml methanol and incubated overnight in the dark at 4 °C to extract flavonoids. The extracted flavonoids were analyzed using LC–ESI–MS/MS (HPLC, Shim-pack UFLC Shimadzu CBM-20A system; MS, Applied Biosystems 4000 Q TRAP) according to the methods described previously^51^.

### SQDG Lipid Analysis

Lipids were extracted and analyzed as described^29^. Briefly, lipids were extracted from leaves with chloroform: methanol (2:1, v/v) containing 0.01% butylated hydroxytoluene (BHT) at room temperature several times until the samples became completely bleached. The resultant lipids were separated on TLC plates using the developing solvent (acetone: toluene: H_2_O = 91:30:7.5, v/v) and were visualized with iodine vapor. The spot corresponding to SQDG was scraped out and was methylated with methanol containing 1% H_2_SO_4_ and 0.05% BHT at 70 °C for 1 h. The SQDG was quantified according to the methods described previously^50^.

### ADPG and UDPG Measurements

Nucleotide sugars were assayed according to the methods described previously^52^. Briefly, fresh leaves with similar age, position and size were incubated in a solvent (chloroform: methanol = 3:7, v/v) at −20 °C for 2 h and were vigorously mixed every 30 min. Nucleotide sugars ADPG and UDPG were then extracted by mixing with an equal volume of ddH_2_O, followed by centrifugation at 13,000 rpm for 5 min. The aqueous phase was transferred to a clean tube and analyzed with HPLC (Shimadzu, SPD-M20A) equipped with C18-RP columns (5 μm, 4.6 × 150 mm). The filtrate was separated by 5% acetonitrile and 95% H_2_O containing 10 mM tetrabutylammonium bromide (Sigma). The flow rate was 1 ml min^−1^, and UV-visible detection was performed at 254 nm.

### Activity Assay for Starch Synthases and Amylase

The activity of soluble SS was detected according to the procedure described previously^53^. In brief, pre-weighed fresh leaf and spikelet tissues were ground in liquid nitrogen, and total protein was extracted with a buffer (50 mM N-(2-hydroxyethyl) piperazine-N’-(2-ethanesulfonic acid)-(HEPES-) NaOH, pH 7.5, 5 mM MgCl_2_, 2 mM DTT, 12% glycerol). For quantitative analysis of soluble SS activity, the extracted proteins (100 μg) were added to a reaction buffer (50 mM HEPES-NaOH, pH 7.5, 1.6 mM ADPG, 15 mM DTT, 1 mg ml^−1^ amylopectin) with or without apigenin 7-O-Glc. After incubation at 37 °C for 20 min, the reaction was terminated in boiling water and was cooled to room temperature. The produced ADP was further measured by incubating with an equal volume of ADP-ATP reaction buffer (50 mM HEPES-NaOH, pH 7.5, 4 mM phosphoenolpyruvate [Sangon, Shanghai], 200 mM KCl, 100 mM MgCl_2_, 1.5 U pyruvate kinase [Sigma]) at 37 °C for 30 min. The ATP product was measured with a Luminescent Kinase Assay Kit according to the manufacturer’s instructions (Promega). The measurement of GBSS activity was similar to that of soluble SS, except for using starch granules as a reaction primer. The SBE activity was assayed according to the procedure described previously^54^. The amylase activity assay was performed as described previously^55^.

## Electronic supplementary material

SQD2.2 supplementary figures
