# Supplementary material for: The Sulfoquinovosyltransferase-like Enzyme SQD2.2 is Involved in Flavonoid Glycosylation, Regulating Sugar Metabolism and Seed Setting in Rice
Source: Sci Rep. 2017 Jul 5;7:4685. doi: 10.1038/s41598-017-04002-2 (PMC5498572; doi:10.1038/s41598-017-04002-2)
Supplement: Supplementary file 1 — SQD2.2 supplementary figures [file 41598_2017_4002_MOESM1_ESM.pdf]

The Sulfoquinovosyltransferase-like Enzyme SQD2.2 is Involved in Flavonoid Glycosylation, Regulating Sugar Metabolism and Seed Setting in Rice

Xinqiao Zhan, Qingwen Shen, Xuemin Wang, Yueyun Hong*

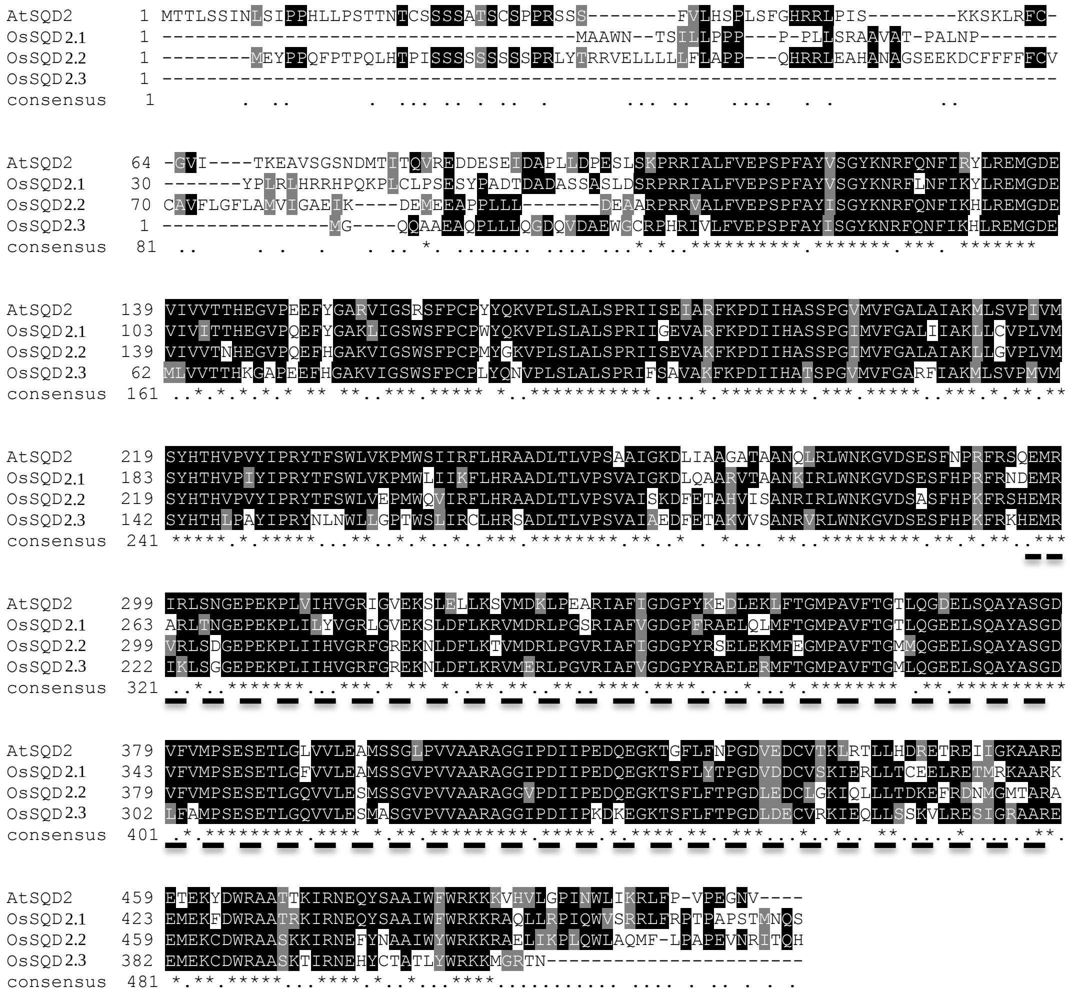

Figure S1. The amino acid sequence alignments of AtSQD2 orthologous proteins using ClustaX program. OsSQD2.1, OsSQD2.2, and OsSQD2.3 share 72%, 67% and 68% amino acid sequence similarity to AtSQD2. The glycosyltransferases domain is underlined. At, *Arabidopsis thaliana*, Os, *Oryza stain*. AtSQD2, AT5g01220; OsSQD2.1, Os07g01030; OsSQD2.2, Os01g04920; OsSQD2.3, Os03g15840.

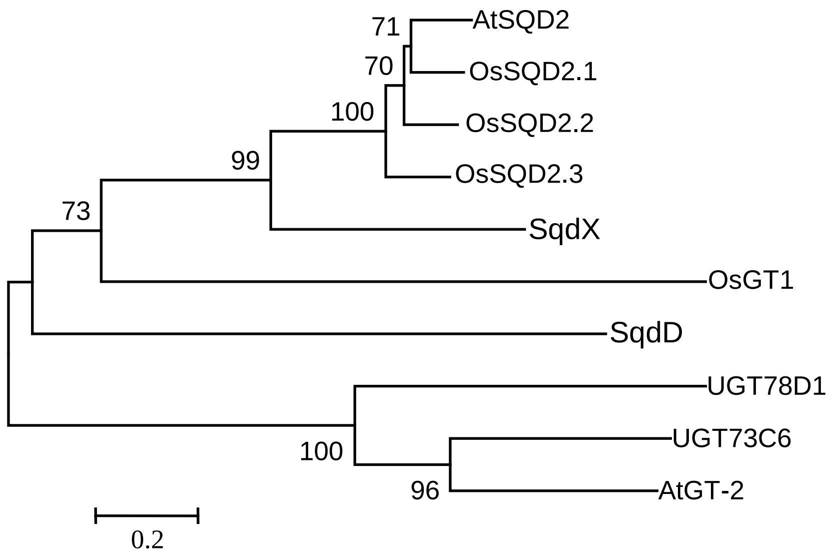

Figure S2. Phylogenetic analysis of rice SQD2.2 orthologs inplants and its relatives. The amino acid sequences of rice SQD2.2 orthologous proteins were aligned using the ClustalX program and were analyzed using the neighbor-joining method, with genetic distance calculated by MEGA5 software. The bar indicates substitutions per site. Bootstrap values (1000 replicates) above 70% are shown on the branches. The amino acid sequences and conserved domain of SQD2.2 orthologous proteins are shown in Figure S1. AtGT-2, At4g34138; OsGT1, Os01g15780; AtSQD2, AT5g01220; OsSQD2.1, Os07g01030; OsSQD2.2, Os01g04920; OsSQD2.3, Os03g15840; AtUGT78D1, AT1G30530; AtUGT73C6, AT2G36790; SqdX, *Synechococcus elongatus* PCC7924 (AAF14309); SqdD, *Rhodobacter sphaeroides* (ABA78725).

```
SQD2.2    1 ATGGAATATCCCCCACAATTCCCTACTCCCCAACTCCACACTCCAATCTCTTCCTCCTCTTCTTCTTCTTCCTCGCCTCG
Seq-R     1 --------------------------------------------------------------------------------

SQD2.2   81 CTTATATACGCGGCGTGTCGAGCTCCTCCTCCTCCTCTTCCTTGCTCCACCACAACACCGTCGACTCGAGGCGCACGCAA
Seq-R     1 --------------------------------------------------------------------------------

SQD2.2  161 ACGCCGGTAGCGAGGAGAAGGATTGCTTCTTCTTTTTTTTTTGTGTGTGTGCTGTGTTCTTGGGTTTCTTGGCAATGGTG
Seq-R     1 --------------------------------------------------------------------------------

SQD2.2  241 ATTGGGGCCGAGATCAAGGACGAGATGGAGGAGGCGCCGCCGCTGCTGCTCGACGAGGCCGCGCGCCCTCGCCGCGTCGC
Seq-R     1 --------------------------------------------------------------------------------

SQD2.2  321 GCTCTTCGTCGAGCCGTCTCCGTTCGCTTACATCTCTGGGTACAAGAACCGGTTTCAGAACTTCATCAAGCACTTGCGCG
Seq-R     1 --------------------------------------------------------------------------------

SQD2.2  401 AAATGGGGGATGAGGTGATTGTTGTGACCAACCATGAGGGGGTTCCCCAAGAATTCCATGGTGCCAAGGTTATTGGTTCA
Seq-R     1 --------------------------------------------------------------------------------

SQD2.2  481 TGGAGCTTTCCCTGTCCAATGTATGGAAAAGTTCCTCTCTCGCTGGCACTCAGTCCTAGGATTATTTCGGAGGTTGCAAA
Seq-R     1 -----------------------------------------------------------------TTCGGAGGTTGCAAA

SQD2.2  561 GTTCAAGCCTGACATCATTCATGCATCCTCACCTGGAATTATGGTTTTTGGGGCACTTGCTATTGCTAAACTGCTCGGTG
Seq-R    16 GTTCAAGCCTGACATCATTCATGCATCCTCACCTGGAATTATGGTTTTTGGGGCACTTGCTATTGCTAAACTGCTCGGTG

SQD2.2  641 TCCCTCTAGTGATGTCCTATCACACCCATGTCCCAGTATACATTCCAAGATATACATTTAGCTGGCTTGTAGAGCCAATG
Seq-R    95 TCCCTCTAGTGATGTCCTATCACACCCATGTCCCAGTATACATTCCAAGATATACATTTAGCTGGCTTGTAGAGCCAATG

SQD2.2  721 TGGCAAGTCATAAGGTTCCTTCATAGAGCTGCTGATCTAACATTAGTGCCATCTGTTGCTATCAGCAAAGATTTTGAAAC
Seq-R   175 TGGCAAGTCATAAGGTTCCTTCATAGAGCTGCTGATCTAACATTAGTGCCATCTGTTGCTATCAGCAAAGATTTTGAAAC

SQD2.2  801 TGCCCATGTTATATCAGCTAATAGAATACGCCTTTGGAACAAAGGTGTTGATTCAGCCAGTTTCCATCCCAAGTTCCGCA
Seq-R   255 TGCCCATGTTATATCAGCTAATAGAATACGCCTTTGGAACAAAGGTGTTGATTCAGCCAGTTTCCATCCCAAGTTCCGCA

SQD2.2  881 GTCACGAAATGCGAGTTAGACTAAGTGACGGTGAGCCTGAAAAACCATTGATAATCCATGTAGGACGCTTTGGGCGTGAA
Seq-R   335 GTCACGAAATGCGAGTTAGACTAAGTGACGGTGAGCCTGAAAAACCATTGATAATCCATGTAGGACGCTTTGGGCGTGAA

SQD2.2  961 AAGAACTTGGATTTTCTGAAAACGGTAATGGATAGGCTGCCTGGAGTAAGAATTGCATTTATTGGAGATGGACCATACAG
Seq-R   415 AAGAACTTGGATTTTCTGAAAACGGTAATGGATAGGCTGCCTGGAGTAAGAATTGCATTTATTGGAGATGGACCATACAG

SQD2.2 1041 GAGTGAGCTGGAGAAGATGTTTGAGGGGATGCCTGCCGTGTTCACTGGAATGATGCAAGGCGAAGAGCTATCACAGGCAT
Seq-R   495 GAGTGAGCTGGAGAAGATGTTTGAGGGGATGCCTGCCGTGTTCACTGGAATGATGCAAGGCGAAGAGCTATCACAGGCAT

SQD2.2 1121 ATGCCAGCGGTGATGTTTTCGTGATGCCCTCGGAGTCCGAAACACTTGGTCAAGTAGTCCTGGAGTCCATGTCATCTGGA
Seq-R   575 ATGCCAGCGGTGATGTTTTCGTGATGCCCTCGGAGTCCGAAACACTTGGTCAAGTAGTCCTGGAGTCCATGTCATCTGGA

SQD2.2 1201 GTCCCGGTCGTTGCAGCTCGTGCTGGTGGTGTTCCTGATATAATTCCAGAAGATCAGGAAGGGAAGACCAGCTTCCTGTT
Seq-R   655 GTCCCGGTCGTTGCAGCTCGTGCTGGTGGTGTTCCTGATATAATTCCAGAAGATCAGGAAGGGAAGACCAGCTTCCTGTT

SQD2.2 1281 CACCCCAGGAGACCTCGAAGACTGTCTTGGCAAGATTCAGCTATTGCTGACGGACAAGGAATTCAGAGACAACATGGGGA
Seq-R   735 CACCCCAGGAGACCTCGAAGACTGTCTTGGCAAGATTCAGCTATTGCTGACGGACAAGGAATTCAGAGACAACATGGGGA

SQD2.2 1361 TGACGGCTAGAGCCGAGATGGAGAAGTGCGACTGGAGAGCAGCTTCCAAGAAGATCCGCAACGAGTTCTACAATGCTGCG
Seq-R   815 TGACGGCTAGAGCCGAGATGGAGAAGTGCGACTGGAGAGCAGCTTCCAAGAAGATCCGCAACGAGTTCTACAATGCTGCG

SQD2.2 1441 ATCTGGTACTGGAGGAAGAAGCGCGCAGAATTGATCAAACCGTTGCAGTGGCTGGCGCAGATGTTCCTACCAGCACCTGA
Seq-R   895 ATCTGGTACTGGAGGAAGAAGCGCGCAGAATTGATCAAACCGTTGCAGTGGCTGGCGCAGA-------------------

SQD2.2 1521 GGTCAACAGAATCACACAACACTGA
Seq-R       -------------------------
```

Figure S3. The sequencing result of the PCR bands in Figure 1A. Seq-R indicates the sequencing result using the vector-specific primer.

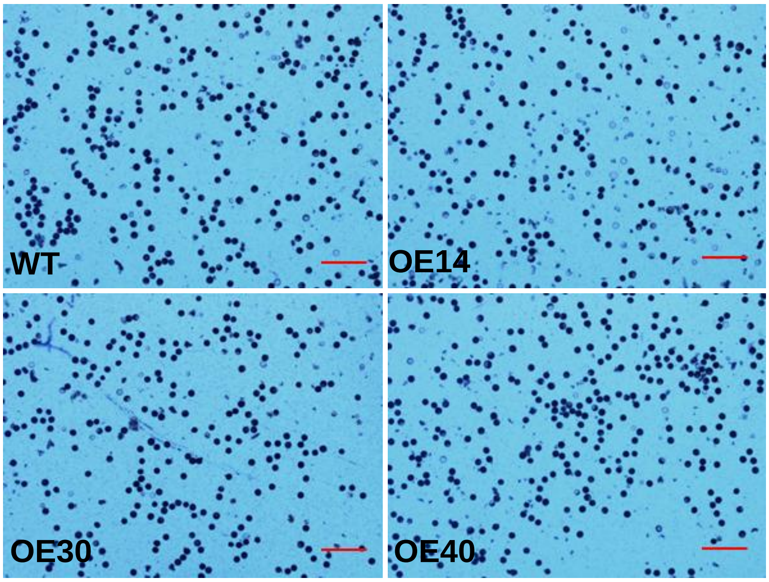

Figure S4. The I_2-KI stained pollen grains of wild-type and *SQD2.2*-OE plants at anthesis stage. Bars = 400 µm.

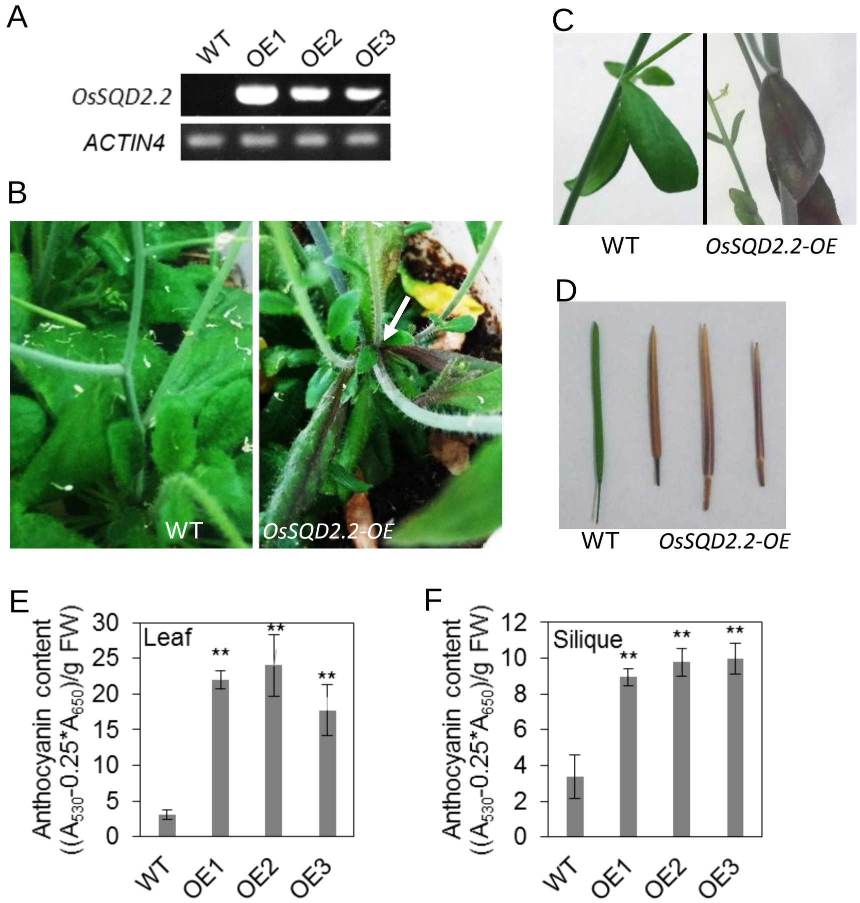

Figure S5. Overexpression of rice *SQD2.2* resulted in flavonoid accumulation in Arabidopsis. (**A**) Rice *SQD2.2* is overexpressed in Arabidopsis and the expression level was confirmed by semi-quantitative RT-PCR. (**B**) to (**D**) Overexpression of *SQD2.2* enhanced the purple color in leaves, stems, and siliques of Arabidopsis. (**E**) and (**F**) Overexpression of *SQD2.2* conferred the anthocyanin accumulation in leaves and siliques of Arabidopsis. Values are the mean ± SD (n = 3). Student's t test, * $P < 0.05$; ** $P < 0.01$.
